# A nationwide study on the current treatment status and natural prognosis of hepatocellular carcinoma in elderly

**DOI:** 10.1038/s41598-023-41771-5

**Published:** 2023-09-04

**Authors:** Jeong-Ju Yoo, Jayoun Lee, Gi Hong Choi, Min Woo Lee, Dong Ah Park

**Affiliations:** 1 Department of Internal Medicine, Soonchunhyang University Bucheon Hospital, Bucheon, South Korea; 2 Division of Healthcare Technology Assessment Research, National Evidence-based Healthcare Collaborating Agency (NECA), 173 Toegye-ro, Jung-gu, Seoul, 04554 South Korea; 3 Department of General Surgery, Yonsei University School of Medicine, Seoul, South Korea; 4 Department of Radiology, Samgsung Medical Center, Sungkyunkwan University, Seoul, South Korea

**Keywords:** Hepatology, Gastroenterology

## Abstract

The aim of this study was to identify the treatment status and natural prognosis of hepatocellular carcinoma (HCC) patients aged 65 years or older in Korea. We analyzed 3,492 patients’ data from the liver cancer stage of the Central Cancer Registry of National Cancer Center. The most common etiology of HCC was hepatitis B (32.7%), followed by hepatitis C. 2624 patients (69.2%) received first-line active treatment for HCC. The most frequently selected treatment was transarterial chemoembolization (TACE), followed by surgical resection and radiofrequency ablation (RFA). The proportion of patients receiving supportive care increased with age. Second-line treatment was performed in only 36.7% of cases, with all others choosing supportive care. Among the various treatments, liver transplantation was found to have the greatest effect in reducing the risk of death (HR [hazard ratio] 0.164, 95% CI [confidence interval] 0.061–0.444), followed by resection, RFA, radioembolization, and TACE. A similar pattern was observed when sub-analyzing the age group over 75 years old. The median survival for untreated HCC in Barcelona Clinic Liver Cancer stage 0/A/B/C/D was 3.7 years, 2.3 years, 7.9 months, 3.9 months, and 2.9 months, respectively. This study highlights the current status of elderly patients with HCC in Korea. While the proportion of patients receiving supportive care is high among the elderly, effective treatment can improve their survival rate.

## Introduction

Hepatocellular carcinoma (HCC) is a primary liver malignancy and one of the most common cancers worldwide, constituting a significant public health problem with a notable impact on morbidity and mortality rates^1,2^. Its incidence is increasing and ranks fourth as a leading cause of cancer-related death globally^3,4^. With the aging of the population, there is an anticipated increase in the number of elderly patients diagnosed with HCC due to its higher incidence in this demographic^5^. Moreover, the majority of HCC cases occur in patients over the age of 60. In developed countries, the incidence of HCC among the elderly has been increasing over the past few decades, partly due to the prevalence of risk factors such as chronic hepatitis B or C infection, alcohol consumption, and non-alcoholic fatty liver disease^6,7^. Therefore, understanding the current status of HCC treatment in the elderly population is of utmost importance.

The treatment options for HCC depend on several factors, including the cancer stage, the patient's overall health, and other individual factors^8^. Surgery is typically the preferred treatment for early-stage HCC^9^. However, elderly patients may be more vulnerable to the side effects of surgery. Liver transplantation is another option, but it may not be feasible for elderly patients due to comorbidities and limited organ availability. Radiation therapy, including external beam radiation therapy (EBRT) and brachytherapy, has shown effectiveness in treating HCC, but its efficacy and safety in the elderly population are not well understood. Systemic therapy, such as chemotherapy and targeted therapy, has also shown potential in treating HCC, but its efficacy and safety in the elderly population are still uncertain. Elderly patients may be more susceptible to the side effects of systemic therapy, such as myelosuppression and hepatotoxicity^10^.

There are several gaps in knowledge that need to be addressed regarding the treatment of HCC in the elderly population. First, more studies are needed on the efficacy and safety of HCC treatment in this population. Second, studies should focus specifically on elderly patients with HCC, as most clinical trials exclude patients over the age of 65. Third, studies should evaluate the impact of comorbidities on the efficacy and safety of HCC treatment in the elderly population. Therefore, the aim of this study is to evaluate the current status of HCC treatment in the elderly population, including the efficacy of various treatment modalities. Furthermore, we investigated the natural course of untreated elderly HCC patients.

## Results

### Characteristics of elderly patients with HCC

Table 1 presents the baseline characteristics of the 3492 elderly HCC patients analyzed in this study. The median age of patients was 72 years (interquartile range [IQR]: 68–77), with 14.9% being over 80 years old. The proportion of male patients was 72.14%, and 32% were ex- or current smokers. Only 2.1% of patients were active drinkers at the time of HCC diagnosis. The Eastern cooperative oncology group (ECOG) performance status was difficult to determine in 31.4% of patients, but status 0 was the most common (68.64%), followed by status 1 (20.84%).

**Table 1:** Demographics and other characteristics of patients.

Variable	N = 3492
Age, years (median [IQR])	72 [68, 77]
65–70 years (number, %)	1130 (32.36)
70–75 years (number, %)	1045 (29.93)
75–80 years (number, %)	796 (22.79)
80–85 years (number, %)	392 (11.23)
≥ 85 years (number, %)	129 (3.69)
Sex (number, %)	
Male	2519 (72.14)
Female	973 (27.86)
Body mass index (median [IQR])	23.54 [21.46, 25.80]
Diabetes (number, %)	1,252 (35.85)
Hypertension (number, %)	1888 (54.07)
Ex- or current smoking (number, %)	1137 (32.56)
Active alcohol drinking (number, %)	76 (2.18)
ECOG performance status (number, %)	(n = 2395, missing = 1097)
Status 0	1644 (68.64)
Status 1	499 (2.84)
Status 2	153 (6.39)
Status 3	66 (2.76)
Status 4	33 (1.38)
Hepatitis B virus surface antigen positive (number, %)	1141 (32.67)
Anti-hepatitis C virus antibody positive (number, %)	659 (18.87)
Ascites (number, %)	
None	2677 (76.66)
Mild	510 (14.60)
Moderate to severe	305 (8.73)
Hepatic encephalopathy (number, %)	
None	3398 (97.31)
Grade I–II	77 (2.21)
Grade III–IV	17 (0.49)
Child–pugh score	(n = 3401, missing = 91)
Class A (number, %)	2471 (72.65)
Class B (number, %)	794 (23.35)
Class C (number, %)	136 (4.00)
MELD score (median [IQR])	9 [7, 11]
MELD-Na score (median [IQR])	10 [8, 14]
Laboratory findings (median [IQR])	
Serum albumin (g/dL)	3.7 [3.2, 4.1]
ALT (IU/mL)	31 [20, 50]
Total bilirubin (mg/dL)	0.9 [0.6, 1.3]
PT INR	1.10 [1.03, 1.20]
Platelet (× 10^3^/μL)	147 [102, 205]
Serum creatinine (mg/dL)	0.9 [0.7, 1.1]
Alpha-fetoprotein (ng/dL)	19.4 [4.7, 310.9]
PIVKA-II (mAU/mL)	104.5 [27.0, 1360.0]
Number of tumors (number, %)	
1	2127 (60.91)
2	551 (15.78)
3	143 (4.10)
4	62 (1.78)
More than 5	609 (17.44)
Size of tumors (cm) (median [IQR])	3.7 [2.0, 4.7]
Huge tumor size (more than 10 cm) (number, %)	573 (16.41)
Invasion of hepatocellular carcinoma (any of the following) (number, %)	750 (21.48)
Portal vein invasion	635 (18.18)
Hepatic vein invasion	162 (4.64)
Bile duct invasion	94 (2.69)
Hepatic artery invasion	35 (1.00)
Regional lymph node metastasis (number, %)	202 (5.78)
Distant metastasis (any of the following) (number, %)	311 (8.91)
Lung metastasis	144 (87.80)
Bone metastasis	85 (77.27)
Lymph node metastasis	79 (75.24)
Others	77 (75.49)
Modified UICC stage (number, %)	(n = 3487, missing = 5)
Stage I (number, %)	457 (13.11)
Stage II (number, %)	1402 (40.21)
Stage III (number, %)	982 (28.16)
Stage IV-A (number, %)	337 (9.66)
Stage IV-B (number, %)	309 (8.86)
Barcelona Clinic Liver Cancer stage (number, %)	(n = 2965, missing = 527)
Stage 0 (number, %)	211 (7.11)
Stage A (number, %)	740 (24.96)
Stage B (number, %)	641 (21.62)
Stage C (number, %)	1154 (38.92)
Stage D (number, %)	219 (7.39)

IQR, interquartile range; ECOG, Eastern Cooperative Oncology Group; MELD, Model For End-Stage Liver Disease; ALT, alanine aminotransferase; PT INR, prothrombin time International Normalized Ratio; PIVKA, protein induced by vitamin K absence; UICC, The Union for International Cancer Control.

The etiology of liver disease was chronic hepatitis B (evaluated by hepatitis B surface antigen positivity) in 32.7% of patients, while 18.9% had chronic hepatitis C, evaluated by anti-hepatitis C virus (HCV) positivity. Ascites was present in 23.3% of patients. 72.65% of the patients were classified as Child–Pugh class A with well-preserved liver function, while 27.35% were Child–Pugh class B or higher. The median model for end-stage liver disease (MELD) score was 9 (IQR 7–11), and the median alpha-fetoprotein (AFP) was 19.4 ng/dL (IQR 4.7–310.9).

### Characteristics of HCC in the elderly

**Figure 1:**
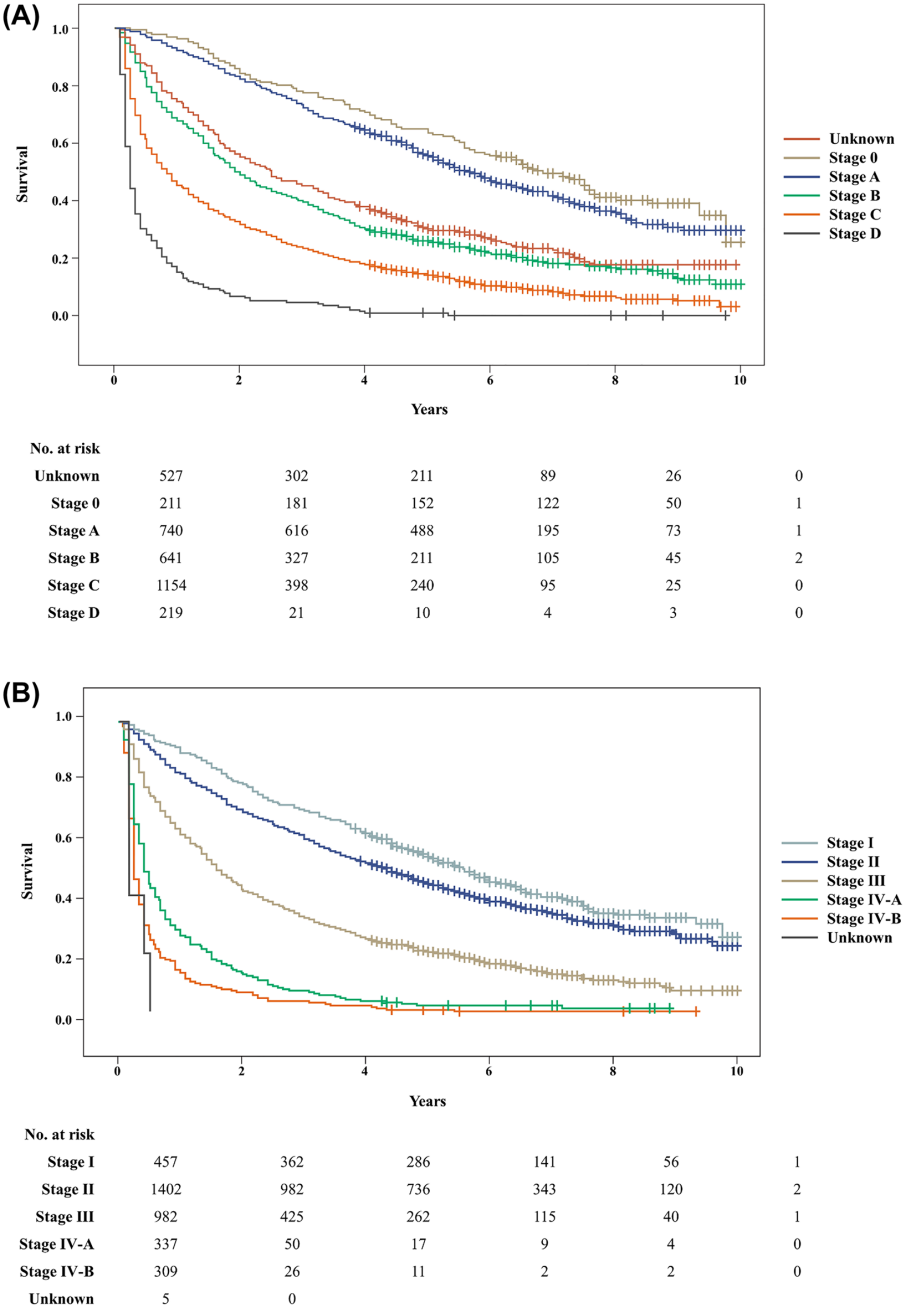
Survival analysis. (**A**) Barcelona Clinic Liver Cancer (BCLC) stage, (**B**) modified the Union for International Cancer Control (UICC) stage.

The characteristics of HCC in the elderly are presented in Table 1. Regarding the number of tumors, 60.91% of the patients had a single tumor, and 17.44% had multiple tumors of 5 or more. The incidence of HCC invasion to the hepatic artery, portal vein, or bile duct was 21.48%, with portal vein invasion being the most common. Distant metastasis was observed in 8.91% of the patients, and lung metastasis was the most common. The modified Union for International Cancer Control (mUICC) stage distribution was as follows: stage II was the most common at 40.21%, followed by stage III and stage I. With respect to the Barcelona Clinic Liver Cancer (BCLC) stage, stage C was the most common at 38.92%, followed by stage A (24.96%) and stage B (21.62%). When classified into BCLC stage (Fig. 1A) or mUICC stage (Fig. 1B), the survival rate tended to significantly decrease as the stage increased (all log-rank p < 0.001). The life table of the patients is presented in Supplementary Table 1.

### Choice of first-line treatment depending on age and cancer stage

Among a total of 3492 elderly patients, 2624 patients (69.2%) received first-line active treatment for HCC, while 868 patients chose supportive care without treatment. The treatment methods were in the order of transarterial chemoembolization (TACE) (1431 patients, 54.53%), surgical resection (563 patients, 21.46%), radiofrequency ablation (RFA) (387 patients, 14.75%), chemotherapy (176 patients, 6.71%), radiation therapy (38 patients, 1.45%), radioembolization (20 patients, 0.76%), and liver transplant (9 patients, 0.34%).

We investigated how first-line treatment for HCC differed by age (Table 2, Supplementary Fig. 1). Although the proportion decreased with age, TACE was the most frequently selected treatment method in all age groups. Moreover, as age increased, the proportion of supportive care gradually increased. Supportive care without active treatment for HCC was chosen by 15.84% of those aged 65–70 years, but 53.49% of those aged 85 years or older. On the other hand, the proportion of surgical resection or RFA gradually decreased with increasing age. Finally, in the case of liver transplantation, there were no cases of liver transplantation in those over 75 years of age.

**Table 2:** Treatment status by age and stage.

By age																	
Years	Supportive care		Surgical resection		RFA		TACE		Chemotherapy		Radiation therapy		Radioembolization		Transplantation		Total
	n	(%)	n	(%)	n	(%)	n	(%)	n	(%)	n	(%)	n	(%)	n	(%)	
65–70	179	(15.84)	263	(23.27)	152	(13.45)	456	(40.35)	54	(4.78)	15	(1.33)	3	(0.27)	8	(0.71)	1130
70–75	214	(20.48)	191	(18.28)	118	(11.29)	461	(44.11)	47	(4.50)	7	(0.67)	6	(0.57)	1	(0.10)	1045
75–80	249	(31.28)	80	(10.05)	88	(11.06)	319	(40.08)	47	(5.90)	9	(1.13)	4	(0.50)	0	(0.00)	796
80–85	157	(40.05)	27	(6.89)	23	(5.87)	155	(39.54)	22	(5.61)	4	(1.02)	4	(1.02)	0	(0.00)	392
≥ 85	69	(53.49)	2	(1.55)	6	(4.65)	40	(31.01)	6	(4.65)	3	(2.33)	3	(2.33)	0	(0.00)	129
Total	868	(24.86)	563	(16.12)	387	(11.08)	1431	(40.98)	176	(5.04)	38	(1.09)	20	(0.57)	9	(0.26)	3492

By tumor stage																	
mUICC stage	Supportive care		Surgical resection		RFA		TACE		Chemotherapy		Radiation therapy		Radioembolization		Transplantation		Total
	n	(%)	n	(%)	n	(%)	n	(%)	n	(%)	n	(%)	n	(%)	n	(%)	
I	38	(8.32)	56	(12.25)	178	(38.95)	177	(38.73)	0	(0.00)	5	(1.09)	1	(0.22)	2	(0.44)	457
II	217	(15.48)	404	(28.82)	168	(11.98)	568	(40.51)	20	(1.43)	9	(0.64)	14	(1.00)	2	(0.14)	1402
III	270	(27.49)	91	(9.27)	34	(3.46)	524	(53.36)	45	(4.58)	8	(0.81)	5	(0.51)	5	(0.51)	982
IV-A	162	(48.07)	9	(2.67)	5	(1.48)	112	(33.23)	44	(13.06)	5	(1.48)	0	(0.00)	0	(0.00)	337
IV-B	177	(57.28)	3	(0.97)	2	(0.65)	49	(15.86)	67	(21.68)	11	(3.56)	0	(0.00)	0	(0.00)	309
Unknown	4	(80.00)	0	(0.00)	0	(0.00)	1	(20.00)	0	(0.00)	0	(0.00)	0	(0.00)	0	(0.00)	5

BCLC stage	Supportive care		Surgical resection		RFA		TACE		Chemotherapy		Radiation therapy		Radioembolization		Transplantation		Total
	n	(%)	n	(%)	n	(%)	n	(%)	n	(%)	n	(%)	n	(%)	n	(%)	
Stage 0	12	(5.69)	24	(11.37)	93	(44.08)	82	(38.86)	0	(0.00)	0	(0.00)	0	(0.00)	0	(0.00)	211
Stage A	40	(5.41)	218	(29.46)	150	(20.27)	322	(43.51)	4	(0.54)	4	(0.54)	2	(0.27)	0	(0.00)	740
Stage B	151	(23.56)	131	(20.44)	6	(0.94)	315	(49.14)	18	(2.81)	5	(0.78)	12	(1.87)	3	(0.47)	641
Stage C	388	(33.62)	104	(9.01)	61	(5.29)	436	(37.78)	139	(12.05)	24	(2.08)	2	(0.17)	0	(0.00)	1154
Stage D	153	(69.86)	2	(0.91)	7	(3.20)	47	(21.46)	5	(2.28)	1	(0.46)	0	(0.00)	4	(1.83)	219
Unknown	124	(23.53)	84	(15.94)	70	(13.28)	229	(43.45)	10	(1.90)	4	(0.76)	4	(0.76)	2	(0.38)	527

RFA, radiofrequency ablation; TACE, transarterial chemoembolization; n, number; UICC, The Union for International Cancer Control; BCLC, Barcelona Clinic Liver Cancer.

Next, we analyzed which treatment was selected depending on the stage of HCC. Based on the mUICC stage, TACE was the most frequently selected therapy for stages I, II, and III. In stage I, RFA followed TACE in 38.73% of cases, and in stage II, surgical resection followed TACE in 28.82% of cases. In stage IV-A or IV-B, supportive care was the most common choice, and when treatment was performed, TACE was the most common in stage IV-A. In stage IV-B, systemic chemotherapy was performed more frequently than TACE. Based on the BCLC stage, RFA was the most selected treatment in stage 0, while TACE was the most selected treatment in stage A or stage B. On the other hand, in stage D, most of the patients chose supportive care, which accounted for 69.86%.

We analyzed whether comorbidity influenced treatment choice. The comorbidities collected in the Liver Cancer Stage open data are diabetes and hypertension information. As a result of the analysis, diabetes did not affect treatment choice (p = 0.336), whereas hypertension had some effect on treatment choice (p = 0.035) (Supplementary Table 2).

### Choice of second-line treatment

We investigated the second-line treatment choices in elderly patients with HCC (Table 3). Out of 2624 elderly patients who received first-line active treatment for HCC, 1343 patients (51.18%) opted for supportive care without second-line treatment. Thus, among the total 3492 patients, only 1281 cases (36.68%) received both first- and second-line treatments.

**Table 3:** First- and second-line treatment of elderly patients with hepatocellular carcinoma.

		Second-line treatment																		Total
		Supportive care		Surgical resection		RFA		TACE		Chemotherapy		Radiation therapy		Radioembolization		Transplantation		Others		
		n	%	n	%	n	%	n	%	n	%	n	%	n	%	n	%	n	%	
First-line treatment	Supportive care	868	100.00																	868
	Surgical resection	378	67.14	10	1.78	27	4.80	106	18.83	31	5.51	6	1.07					5	0.89	563
	RFA	242	62.53	2	0.52	70	18.09	61	15.76			6	1.55					6	1.55	387
	TACE	543	37.95	34	2.38	103	7.20	624	43.61	31	2.17	55	3.84	3	0.21	6	0.42	32	2.24	1431
	Chemotherapy	141	80.11	1	0.57	1	0.57	4	2.27	13	7.39	15	8.52					1	0.57	176
	Radiation therapy	25	65.79	1	2.63			2	5.26	7	18.42	3	7.89							38
	Radioembolization	6	30.00	2	10.00			8	40.00					2	10.00			2	10.00	20
	Transplantation	8	88.89							1	11.11									9

RFA, radiofrequency ablation; TACE, transarterial chemoembolization; n, number; UICC, The Union for International Cancer Control; BCLC, Barcelona Clinic Liver Cancer.

Among the 563 patients who underwent surgical resection as the first treatment, 378 patients (67.14%) chose supportive care as the second-line treatment, and TACE was performed as the second-line treatment in 106 patients (18.83%). Of the 387 patients who underwent RFA as the first-line treatment, 242 patients (62.53%) chose supportive care as the second-line treatment, followed by RFA and TACE. Conversely, out of the 1,431 patients who received TACE as the first treatment, 624 patients (43.61%) underwent TACE again as the second-line treatment.

### Survival rates according to first-line treatment or cancer stage

**Figure 2:**
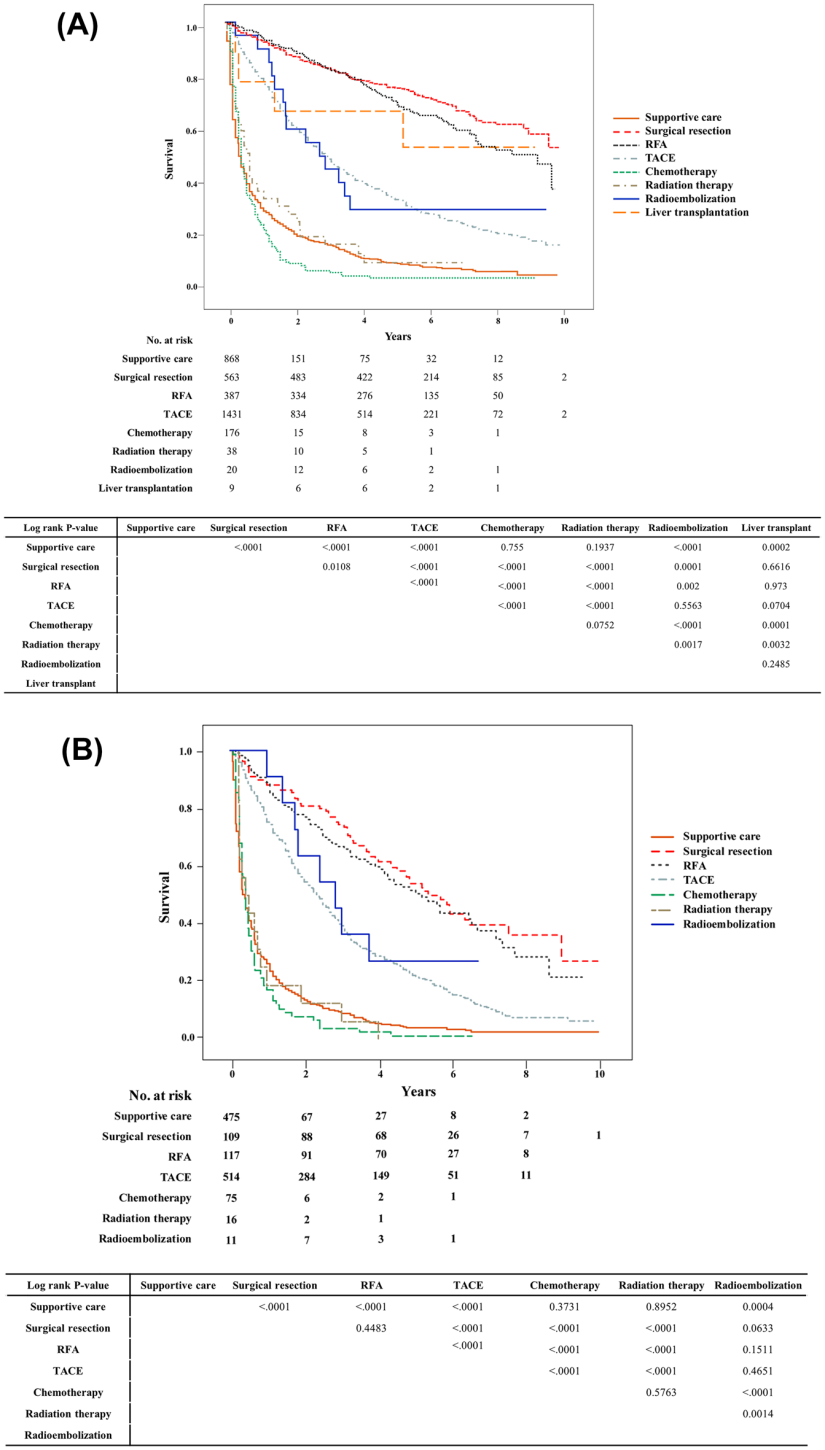
Comparison of overall survival according to treatment method. (**A**) All elderly patients (≥ 65 years), (**B**) very elderly patients (≥ 75 years).

Next, we analyzed the survival rate depending on the first-line treatment method. Survival was significantly higher when surgical resection, liver transplantation, and RFA were selected as the first-line treatment methods, compared to other treatments (Fig. 2A). Although survival rate was lower than the above three methods, TACE or radioembolization showed similar treatment results. On the other hand, supportive care, chemotherapy, and radiotherapy had the poorest survival rate. As a next step, we analyzed whether there was a difference in survival according to the treatment method within the same BCLC stage. Treatment effects were compared in each of the early stage (BCLC stage 0 or A, Supplementary Fig. 2A), intermediate stage (BCLC stage B, Supplementary Fig. 2B), and advanced stage (BCLC stage C, Supplementary Fig. 2C). In the subgroup, the survival rates in the order of surgery, RFA, and TACE showed the same tendency when compared with the whole group. The life table of the patients is presented in Supplementary Table 3.

Sensitivity survival analysis according to first line treatment was performed only for patients aged 75 years or older (Fig. 2B). The overall pattern was similar to that of patients older than 65 years. Surgical resection and RFA had the best survival rate, followed by patients who received TACE or radioembolization. Lastly, chemotherapy and radiation therapy showed the poorest survival rates similar to supportive care.

### Factors associated with mortality in elderly HCC

Factors related to mortality were analyzed by Cox regression analysis and hazard ratio (HR) statistics were reported (Table 4). In multivariate analysis, the mortality rate was significantly lower when liver transplantation (HR 0.164, 95% CI [confidence interval] 0.061–0.444), surgical resection (HR 0.231, 95% CI 0.195–0.273), RFA (HR 0.296, 95% CI 0.246–0.356), and radioembolization (HR 0.447, 95% CI 0.251–0.795) were performed as first-line treatment methods. Additionally, mortality was significantly higher in cases with advanced age, low body mass index (BMI), diabetic comorbidity, performance status of 2 or higher, poor underlying liver function (Child–Pugh class B or C, MELD ≥ 10), high cancer stage, or low platelet count (< 150 × 10^3^/μL). Additionally, cox regression analysis was performed on factors related to survival in patients aged 75 years or older (Supplementary Table 4). Risk factors were similar as those of patients aged 65 years or older. Treatment option (surgical resection, TACE, RFA, radioembolization), high BMI, preserved liver function, single tumor, small tumor less than 3 cm was associated with prolonged survival.

**Table 4:** Factors affecting overall mortality in elderly patients with hepatocellular carcinoma.

Variables	Univariable		Multivariable	
	HR (95% CI)	*P*	HR (95% CI)	*P*
Treatment option (first-line)				
Supportive care	1 (ref)		1 (ref)	
Surgical resection	0.114 (0.099–0.133)	< 0.001	0.231 (0.195–0.273)	< 0.001
RFA	0.144 (0.123–0.169)	< 0.001	0.296 (0.246–0.356)	< 0.001
TACE	0.331 (0.302–0.363)	< 0.001	0.503 (0.452–0.560)	< 0.001
Systemic chemotherapy	1.165 (0.987–1.375)	0.707	0.934 (0.775–1.125)	0.472
Radiation therapy	0.808 (0.576–1.134)	0.218	0.848 (0.597–1.203)	0.354
Radioembolization	0.285 (0.168–0.483)	< 0.001	0.447 (0.251–0.795)	0.006
Liver transplantation	0.142 (0.053–0.380)	< 0.001	0.164 (0.061–0.444)	< 0.001
Male sex (vs. female)	1.038 (0.953–1.131)	0.392	1.044 (0.949–1.148)	0.380
Age group				
65–70 years	1 (ref)		1 (ref)	
70–75 years	1.278 (1.154–1.415)	< 0.001	1.116 (1.003–1.242)	0.044
75–80 years	0.735 (1.559–1.930)	< 0.001	1.344 (1.197–1.509)	< 0.001
80–85 years	2.458 (2.163–2.794)	< 0.001	1.646 (1.427–1.898)	< 0.001
≥ 85 years	3.051 (2.506–3.714)	< 0.001	1.470 (1.181–1.830)	< 0.001
High BMI (≥ 25 kg/m^2^)	0.713 (0.654–0.777)	< 0.001	0.824 (0.752–0.903)	< 0.001
Diabetes	1.126 (1.040–1.220)	0.003	1.125 (1.031–1.228)	0.008
Hypertension	0.901 (0.834–0.973)	0.007	1.001 (0.919–1.090)	0.981
ECOG performance status				
Status 0	1 (ref)		1 (ref)	
Status 1	1.603 (1.428–1.799)	< 0.001	1.198 (1.062–1.352)	0.003
Status 2	3.034 (2.543–3.619)	< 0.001	1.396 (1.158–1.684)	< 0.001
Status 3	3.902 (3.012–5.054)	< 0.001	1.171 (0.879–1.561)	0.281
Status 4	9.167 (6.458–13.014)	< 0.001	3.002 (2.078–4.336)	< 0.001
HBsAg-positive	0.766 (0.703–0.834)	< 0.001	0.912 (0.824–1.009)	0.073
Anti-HCV positive	1.069 (0.970–1.178)	0.178	1.018 (0.911–1.136)	0.756
Ascites				
None	1 (ref)			
Mild	2.700 (1.436–2.993)	< 0.001		
Moderate to severe	4.237 (3.734–4.807)	< 0.001		
Child–pugh class				
Class A	1 (ref)			
Class B	2.896 (2.650–3.164)	< 0.001	1.732 (1.558–1.927)	< 0.001
Class C	5.756 (4.809–6.889)	< 0.001	2.530 (2.040–3.138)	< 0.001
MELD score				
< 10	1 (ref)		1 (ref)	
≥ 10	1.985 (1.832–2.151)	< 0.001	1.174 (1.070–1.289)	< 0.001
Number of tumors				
1	1 (ref)		1 (ref)	
More than 2	1.908 (1.765–2.062)	< 0.001	1.194 (1.048–1.362)	0.007
Size of tumors				
< 3 cm	1 (ref)		1 (ref)	
≥ 3 cm	1.746 (1.612–1.891)	< 0.001	1.183 (1.072–1.307)	< 0.001
Portal vein invasion	3.401 (3.111–3.718)	< 0.001	1.389 (1.197–1.613)	< 0.001
Modified UICC stage				
Stage I	1 (ref)		1 (ref)	
Stage II	1.240 (1.080–1.423)	0.002	0.984 (0.841–1.153)	0.844
Stage III	2.437 (2.120–2.802)	< 0.001	1.245 (1.009–1.538)	0.041
Stage IV-A	5.925 (5.019–6.993)	< 0.001	1.762 (1.310–2.371)	< 0.001
Stage IV-B	9.027 (7.614–10.702)	< 0.001	2.422 (1.884–3.113)	< 0.001
BCLC stage				
Stage 0	1 (ref)			
Stage A	1.119 (0.910–1.377)	< 0.001		
Stage B	2.401 (1.962–2.938)	< 0.001		
Stage C	3.811 (3.142–4.622)	< 0.001		
Stage D	9.200 (7.317–11.568)	< 0.001		
Serum albumin				
< 4 g/dL	1 (ref)			
≥ 4 g/dL	0.384 (0.351–0.419)	< 0.001		
Total bilirubin				
< 1 mg/dL	1 (ref)			
≥ 1 mg/dL	1.654 (1.531–1.787)	< 0.001		
Platelet (× 10^3^/μL)				
≥ 150	1 (ref)			
< 150	1.082 (1.002–1.168)	0.044	1.122 (1.026–1.227)	0.011

HR, hazard ratio; CI, confidence interval; RFA, radiofrequency ablation; TACE, trasarterial chemoembolization; BMI, body mass index; ECOG, Eastern Cooperative Oncology Group; HBsAg, hepatitis B virus surface antigen; HCV, hepatitis C virus; MELD, Model For End-Stage Liver Disease; UICC, The Union for International Cancer Control; BCLC, Barcelona Clinic Liver Cancer.

### Natural course of elderly HCC

**Figure 3:**
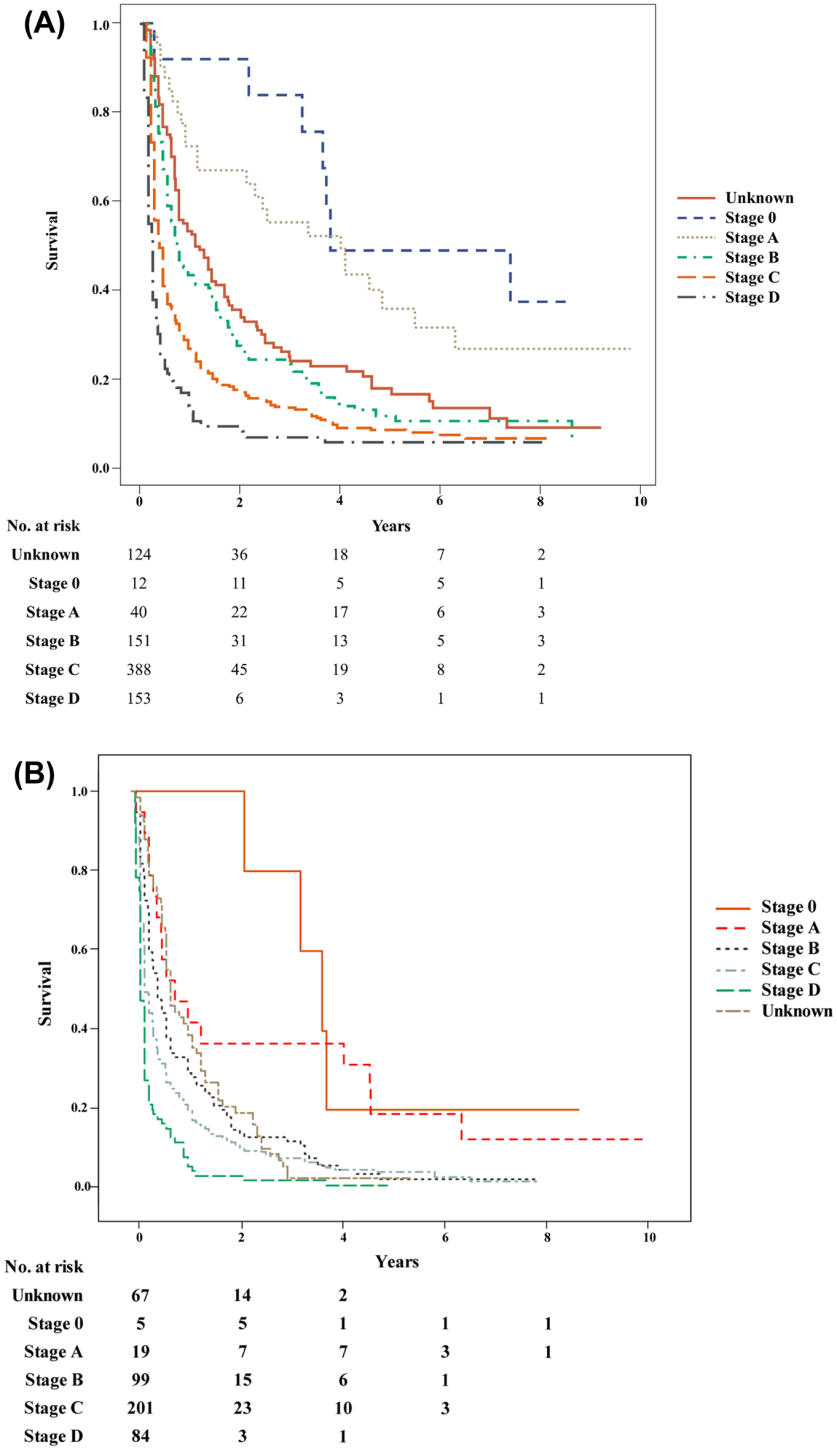
Natural prognosis of untreated hepatocellular carcinoma in elderly. (**A**) All elderly patients (≥ 65 years), (**B**) very elderly patients (≥ 75 years).

In our final analysis, we examined the natural course of HCC in elderly patients who received only supportive care, as subgroups (Fig. 3A). When classified depending on BCLC stage, we found that the median survival values for stages 0, A, B, C, and D were 3.79 years, 2.33 years, 0.66 years, 0.33 years, and 0.24 years, respectively. The 1-year survival rates were 91.7%, 65.0%, 37.1%, 19.6%, and 6.5% in stages 0, A, B, C, and D, respectively. The 3-year survival rates were 83.3%, 45.0%, 15.9%, 8.3%, and 2.6%, respectively. Moreover, the 5-year survival rates were 41.7%, 25.9%, 5.9%, 0.4%, and 0.2% in stages 0, A, B, C, and D, respectively. Similarly, HCC natural prognosis was also evaluated in patients aged 75 years or older (Fig. 3B). Compared to patients aged 65 years or older, the median survival was poorer. Median survival was 3.66 years, 0.83 year, 0.50 year, 0.24 year, and 0.16 year respectively in stages 0, A, B, C, and D of HCC patients aged 75 years or older. The life table of the patients is presented in Supplementary Table 5.

## Discussion

In this study, we investigated the characteristics of elderly HCC, the current status of first-line and second-line treatment, treatment efficacy, and the natural course of HCC in the elderly.

HCC remains an important clinical problem in East Asia, with an incidence approximately 10 times higher than in the West^11^. In fact, HCC occurring in high incidence areas such as East Asia or sub-Saharan Africa accounts for about 80% of HCC worldwide^11^. In South Korea, one of the high incidence areas, there is a recent change in the age pattern of HCC. According to a published study, while the overall incidence of HCC is decreasing, HCC in the elderly is increasing, and it is predicted that by 2028, about 21.3% of HCC patients will be over 80 years of age^5^. Our study has great clinical significance as it investigated the HCC treatment status of elderly individuals in a high incidence area of HCC based on a large cohort.

The first finding of our study is regarding the general characteristics of HCC in the elderly. Previous studies have reported that elderly patients with HCC are characterized by a high proportion of women or non-viral etiology^12,13^. In our study, we confirmed these same characteristics. Compared to the analysis of all age groups of HCC patients in South Korea, we found a higher proportion of women in elderly HCC (27.9% vs. 20.3%) and a higher proportion of HCV patients (18.9% vs. 8.2%), while the proportion of hepatitis B virus (HBV) patients was lower (32.7% vs. 58.1%)^14^. This can be explained by the fact that women have a longer average life expectancy than men, HCC caused by HCV tends to take about 10 years longer than HCC caused by HBV, and the rate of HCC caused by fatty liver is increasing recently^15^. Although HBV is the most important cause of HCC in South Korea, which is an HBV endemic area, the rate of HCC caused by non-viral etiology is expected to increase significantly in the future due to the HBV vaccine and effective antiviral treatment^16^. Furthermore, HCC that develops from fatty liver takes much longer than HCC that develops from viral hepatitis, which suggests that the average age of elderly HCC patients is likely to increase further in the future^17^.

In addition, among elderly patients with HCC, a high proportion had comorbidities such as diabetes (35.9%) or hypertension (54.0%), and many had poor performance status. These factors can greatly impact treatment selection and prognosis. In fact, performance status 1 or 2 are classified as BCLC stage C, while performance status 3 or 4 are classified as stage D. In our study, about one-fourth (24.9%) of elderly HCC patients chose supportive care, which was higher than the 20.2% of non-elderly HCC patients. This suggests that comorbidity or performance status influenced the choice of treatment method. Previous studies have reported that the degree of liver fibrosis in elderly HCC patients was less than that of non-elderly patients^12^. However, in our study, we found no difference in Child–Pugh score or MELD score when compared to a previous study that investigated non-elderly people.

The second finding of our study concerns the treatment options chosen for elderly patients with HCC. TACE was the most frequently selected, followed by surgical resection and RFA, and the proportion of these treatment methods did not show a significant difference from previous studies. This is likely because the characteristics of elderly HCC patients (such as the number of tumors, invasion, metastasis, and stage) were not significantly different from those of non-elderly patients. In clinical practice, the most important factor in selecting a treatment method is the stage of HCC. In our study, the prognosis of elderly HCC patients who underwent treatment was favorable. This finding is consistent with previous reports that while the overall survival of elderly HCC patients is not good, the outcome is similar to that of non-elderly patients when treated^18^. Therefore, the stage and performance status of HCC should be considered in the treatment method rather than the patient's age alone, and treatment should be equally recommended for BCLC stage regardless of age. However, according to the results of multivariate analysis in our study, mortality significantly increased in patients aged 75 years (HR 1.34) or 80 years (HR 1.64) and older, indicating the need for clinical caution in very elderly patients.

Our study presents, for the first time, findings on the selection of second-line treatment for elderly HCC patients. However, supportive care was the most frequently selected second-line option after TACE, indicating that the possibility of implementing second-line treatment in elderly HCC patients is low. Therefore, more attention should be paid to the selection of first-line treatment. In our study, mortality was reduced in the order of liver transplant, surgical resection, RFA, radioembolization, and TACE. Thus, it would be clinically beneficial to select these treatments whenever possible.

Our study also shed light on the natural prognosis of untreated elderly HCC patients. In our study, we found that the median survival of untreated HCC in BCLC stage 0/A/B/C/D was 3.7 years, 2.3 years, 7.9 months, 3.9 months, and 2.9 months, respectively. Notably, the survival rate in BCLC stage B was much lower than the 16 months reported in previous studies^19^. Although there are few reports on the natural course of early stage HCC in the elderly, our study provides valuable information with a result of 2.3–3.7 years. This finding can help patients or their families make informed decisions about whether or not to receive treatment in the future.

Further research is necessary in the following areas. First, our study was conducted prior to the use of effective therapies such as atezolizumab with bevacizumab, so the recent therapies were not reflected. Despite concerns about progressive decline in immune function with increasing age, fortunately, the effects of immunotherapy such as atezolizumab, nivolumab, and pembrolizumab in the elderly were similar to those in the non-elderly patients^20–23^. Age was not a significant factor in overall survival or progression free survival in most studies. In addition, the side effects of immunotherapy in elderly patients were not significantly different from those of non-aged patients, and were mostly manageable when the drug was discontinued^20–23^. However, most of the reference for immunotherapy for the elderly are clinical trial data, and in many cases, there was an age limit in these clinical trials, so real-practice research should be conducted additionally^24^. Therefore, more studies on HCC in the elderly are needed in the era of potent immunotherapy. Second, there is still a lack of prospective studies on elderly HCC. In the future, it is essential to gather more prospective data on this patient group by expanding the age limit in a prospective cohort study or clinical trial of elderly HCC patients. Third, given the high prevalence of comorbidities in elderly HCC patients, various comorbidities may influence on treatment decisions and prognosis. In our study, the effects of hypertension and diabetes on treatment method were analyzed, but other comorbidities were not analyzed due to lack of data. In the future, studies on the effects of various comorbidities on liver cancer treatment are expected to provide useful information to clinicians.

Conclusively, elderly patients with HCC exhibit unique characteristics that significantly influence the choice of treatment approaches. Nonetheless, favorable outcomes are associated with treatments, and thus active treatment should be considered wherever possible. Clinicians should be knowledgeable about the natural course of elderly HCC and treatment effects and strive to select optimal treatment methods for this special group.

## Methods

### Patients

To analyze the current status of medical service use by elderly HCC patients in South Korea, we utilized the Liver Cancer Stage open data (2011–2016), which includes clinical information from the Central Cancer Registry at the National Cancer Center. The data was obtained through a retrospective medical record survey, with samples taken from HCC patients registered at the Central Cancer Registry. The data includes demographic characteristics, diagnosis information, and treatment information of study subjects. The sampling method for Liver Cancer Stage open data involved selecting hospitals to be surveyed by year based on selection criteria and then extracting 10% through phylogenetic sampling. The 13 nationally designated cancer centers considered for the present study were high-ranking hospitals that accounted for over 75% of the number of patients with HCC. Liver cancer stage open data is open to the public by the Korea Central Cancer Registry, a national institution, and anyone can download it through deliberation by submitting an appropriate research proposal. The database can be accessed through the web page https://kccrsurvey.cancer.go.kr/index.do.

The Institutional Review Boards (IRB) of the National Evidence-based Healthcare Collaborating Agency approved the study protocol (IRB number: NECA-IRB21-016, Date of registration: 17-June-2021). Informed consent was waived from the IRB of the National Evidence-based Healthcare Collaborating Agency due to the retrospective design. The study protocol adhered to the ethical guidelines of the World Medical Association Declaration of Helsinki.

The study subjects were selected from patients aged 65 years or older who were registered in the Liver Cancer Stage open data from 2011 to 2016. Cases in which detailed information regarding treatment method classification, imaging diagnosis results, tumor number, gender, etc., was not clear were excluded. Finally, a total of 3492 elderly HCC patients were selected for analysis (Supplementary Fig. 3).

### Data collection

In the Liver Cancer Stage open data, various pieces of information are available, including age, gender, height, weight, comorbidity, cause of liver disease, performance status, blood test results, tumor markers, imaging diagnosis results, major vascular invasion, histological examination, Child–Pugh class, mUICC stage, BCLC stage, first treatment method, and second treatment method. The treatment methods were classified as surgical resection, liver transplantation, RFA, TACE, radioembolization, systemic chemotherapy (sorafenib or cytotoxic chemotherapy such as oxaliplatin-containing regimen), radiation therapy, and supportive treatment only. In addition, as the study was conducted before the introduction of immunotherapeutic agents (e.g. atezolizumab or nivolumab), there were no patients who received immunotherapeutic agents. Percutaneous ethanol injection therapy and hepatic artery chemoinfusion, which had very few subjects (less than 5), were excluded from the analysis. The overall survival was defined as the duration from the first date of liver cancer diagnosis (index date) to the last day of survival.

### Statistical analysis

For the analysis results, the information on the elderly aged 65 years or older was first described, and the information on patients aged 75 years or older was additionally described for sensitivity analysis. We expected that this additional analysis involving a higher age threshold (75 years) will be helpful for clinicians, shedding more light on the impact of treatment options in this specific population.

Continuous baseline characteristics were presented as means (± standard deviations) and compared using Student’s t-test. Categorical characteristics were presented as counts and percentages and compared between groups using the chi-squared test. Cox regression analysis was conducted to evaluate the factors related to mortality. Factors that were significant in univariate analysis (*P* < 0.05) or identified as risk factors in previous studies such as gender or etiology of liver disease were used in multivariate analysis^25,26^. If there are two or more factors that can cause collinearity (e.g. ascites and Child–pugh class), only one of them was included in the multivariate analysis. Statistically significant differences were defined as *P* < 0.05. All statistical analyses were performed using R version 4.3.1 (The R Foundation for Statistical Computing, Vienna, Austria).

### Ethics declarations and Informed consent statement

The Institutional Review Boards (IRB) of the National Evidence-based Healthcare Collaborating Agency approved the study protocol (IRB number: NECA-IRB21-016, Date of registration: 17-June-2021). The study protocol adhered to the ethical guidelines of the World Medical Association Declaration of Helsinki. Informed consent was waived from the IRB of the National Evidence-based Healthcare Collaborating Agency due to the retrospective design.

### Supplementary Information

Supplementary Figures.

Supplementary Tables.

## Data Availability

Liver cancer stage open data is open to the public by the Korea Central Cancer Registry, a national institution, and anyone can download it through deliberation by submitting an appropriate research proposal. The database can be accessed through the web page https://kccrsurvey.cancer.go.kr/index.do.
