# Supplementary material for: A nationwide study on the current treatment status and natural prognosis of hepatocellular carcinoma in elderly
Source: Sci Rep. 2023 Sep 4;13:14584. doi: 10.1038/s41598-023-41771-5 (PMC10477316; doi:10.1038/s41598-023-41771-5)
Supplement: Supplementary file 1 — Supplementary Figures. [file 41598_2023_41771_MOESM1_ESM.docx]

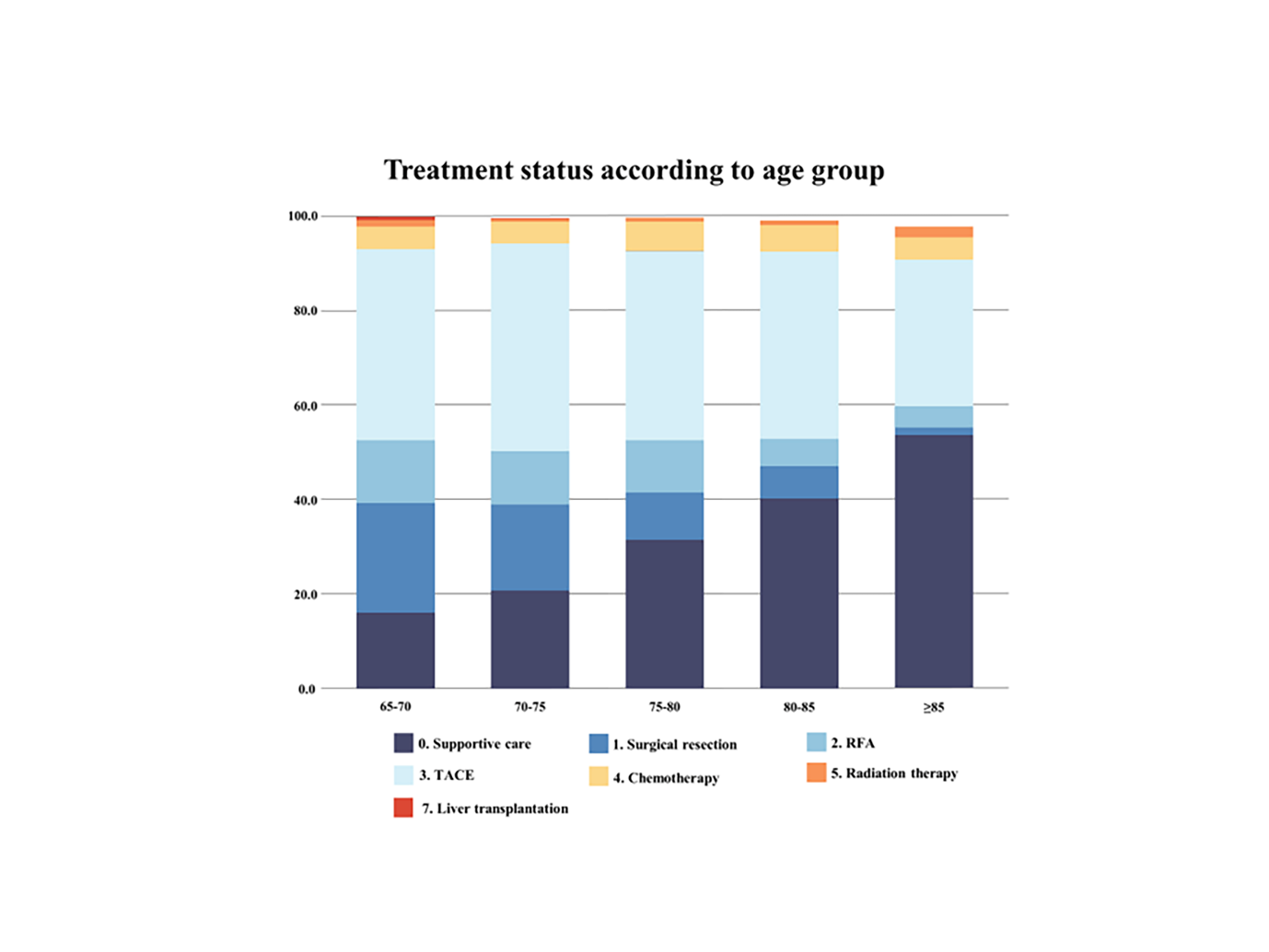


**Supplementary Fig 1.** **First-line treatment option in elderly patients with hepatocellular carcinoma**


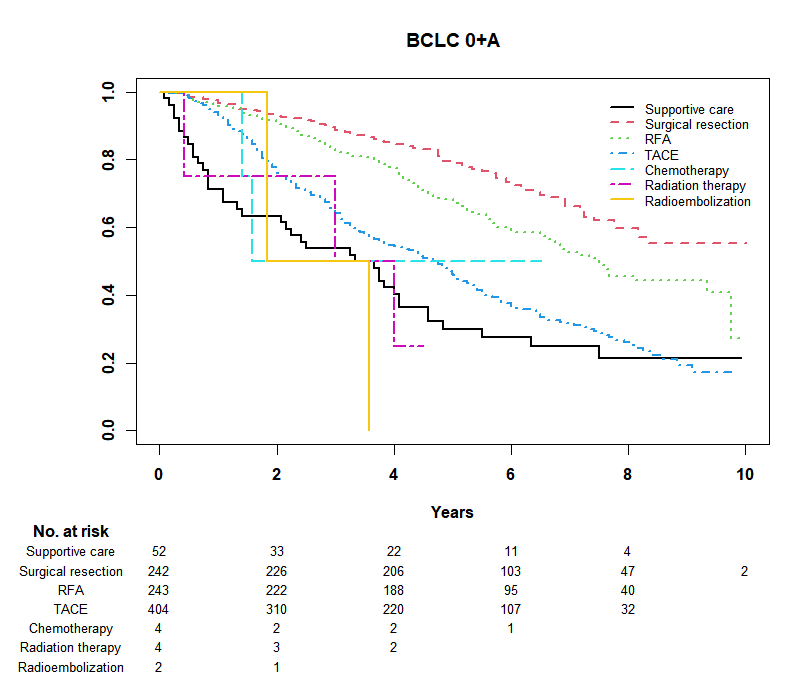


| **Log rank P-value** | **Supportive care** | **Surgical resection** | **RFA** | **TACE** | **Chemotherapy** | **Radiation therapy** | **Radioembolization** | **Liver transplant** |
| --- | --- | --- | --- | --- | --- | --- | --- | --- |
| **Supportive care** |  | <.0001 | <.0001 | <.0001 | 0.755 | 0.1937 | <.0001 | 0.0002 |
| **Surgical resection** |  |  | 0.0108 | <.0001 | <.0001 | <.0001 | 0.0001 | 0.6616 |
| **RFA** |  |  |  | <.0001 | <.0001 | <.0001 | 0.002 | 0.973 |
| **TACE** |  |  |  |  | <.0001 | <.0001 | 0.5563 | 0.0704 |
| **Chemotherapy** |  |  |  |  |  | 0.0752 | <.0001 | 0.0001 |
| **Radiation therapy** |  |  |  |  |  |  | 0.0017 | 0.0032 |
| **Radioembolization** |  |  |  |  |  |  |  | 0.2485 |
| **Liver transplant** |  |  |  |  |  |  |  |  |

**Supplementary Fig 2A. Survival analysis depending on treatment method:** Early stage HCC (BCLC stage 0 or A)


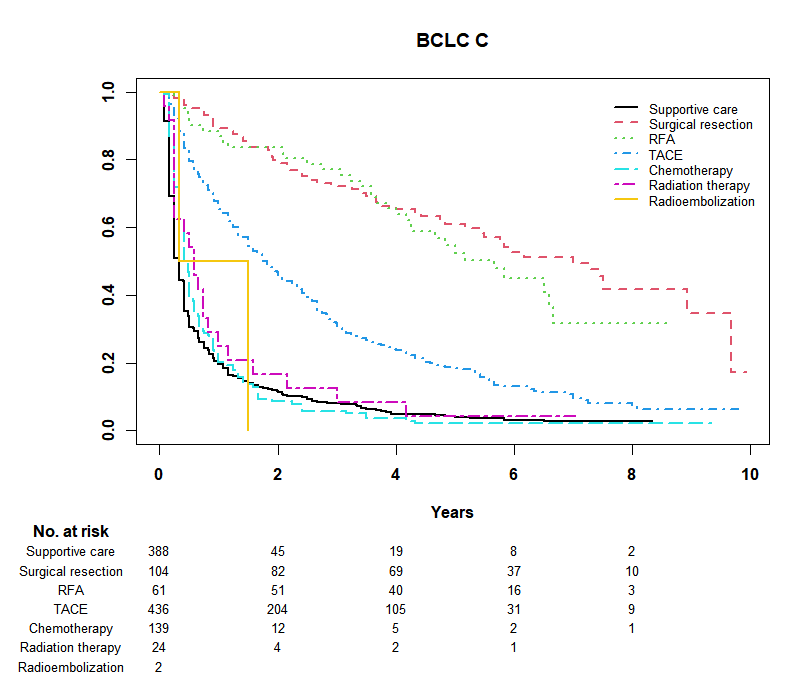

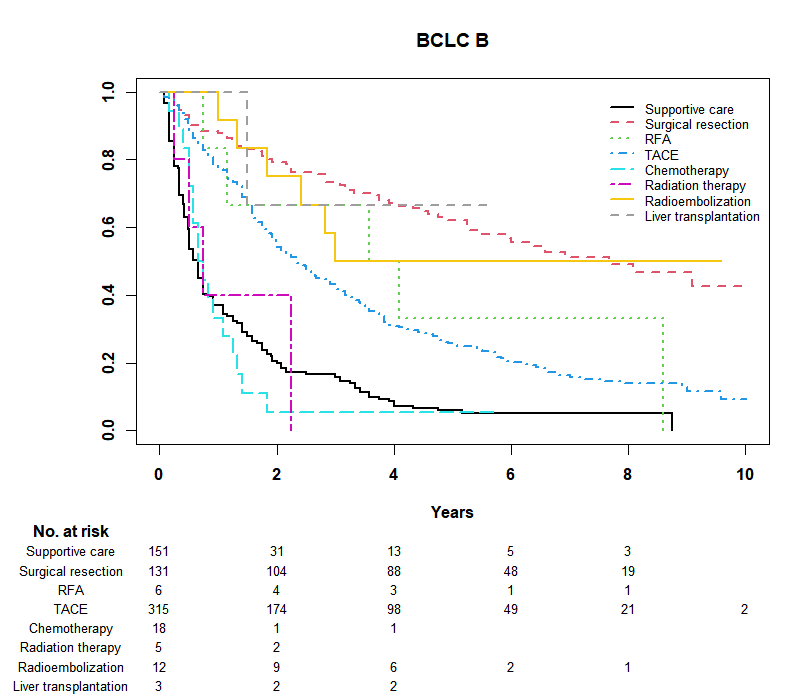


| **Log rank P-value** | **Supportive care** | **Surgical resection** | **RFA** | **TACE** | **Chemotherapy** | **Radiation therapy** | **Radioembolization** | **Liver transplant** |
| --- | --- | --- | --- | --- | --- | --- | --- | --- |
| **Supportive care** |  | <.0001 | 0.0283 | <.0001 | 0.8553 | 0.9652 | <.0001 | 0.0246 |
| **Surgical resection** |  |  | 0.0917 | <.0001 | <.0001 | <.0001 | 0.6127 | 0.8616 |
| **RFA** |  |  |  | 0.6618 | 0.0194 | 0.0343 | 0.4147 | 0.4198 |
| **TACE** |  |  |  |  | <.0001 | 0.0097 | 0.0583 | 0.2209 |
| **Chemotherapy** |  |  |  |  |  | 0.5940 | <.0001 | 0.022 |
| **Radiation therapy** |  |  |  |  |  |  | 0.0013 | 0.0847 |
| **Radioembolization** |  |  |  |  |  |  |  | 0.6952 |
| **Liver transplant** |  |  |  |  |  |  |  |  |

**Supplementary Fig 2B. Survival analysis depending on treatment method:** intermediate stage HCC (BCLC stage B)


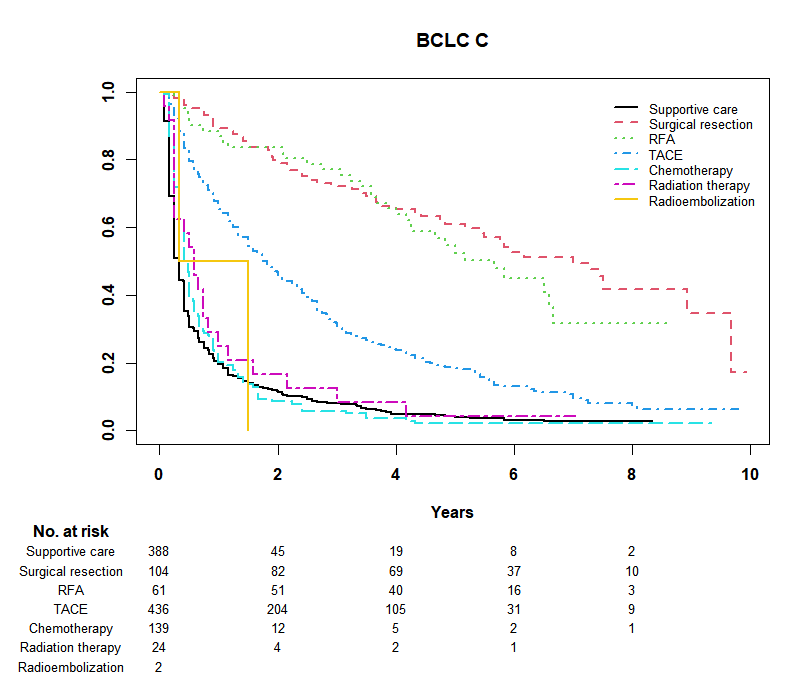


| **Log rank P-value** | **Supportive care** | **Surgical resection** | **RFA** | **TACE** | **Chemotherapy** | **Radiation therapy** | **Radioembolization** |
| --- | --- | --- | --- | --- | --- | --- | --- |
| **Supportive care** |  | <.0001 | <.0001 | <.0001 | 0.1728 | 0.1831 | 0.6688 |
| **Surgical resection** |  |  | 0.3179 | <.0001 | <.0001 | <.0001 | <.0001 |
| **RFA** |  |  |  | <.0001 | <.0001 | <.0001 | 0.0008 |
| **TACE** |  |  |  |  | <.0001 | <.0001 | 0.1351 |
| **Chemotherapy** |  |  |  |  |  | 0.4069 | 0.776 |
| **Radiation therapy** |  |  |  |  |  |  | 0.9924 |
| **Radioembolization** |  |  |  |  |  |  |  |

**Supplementary Fig 2C. Survival analysis depending on treatment method:** advanced stage HCC (BCLC stage C)


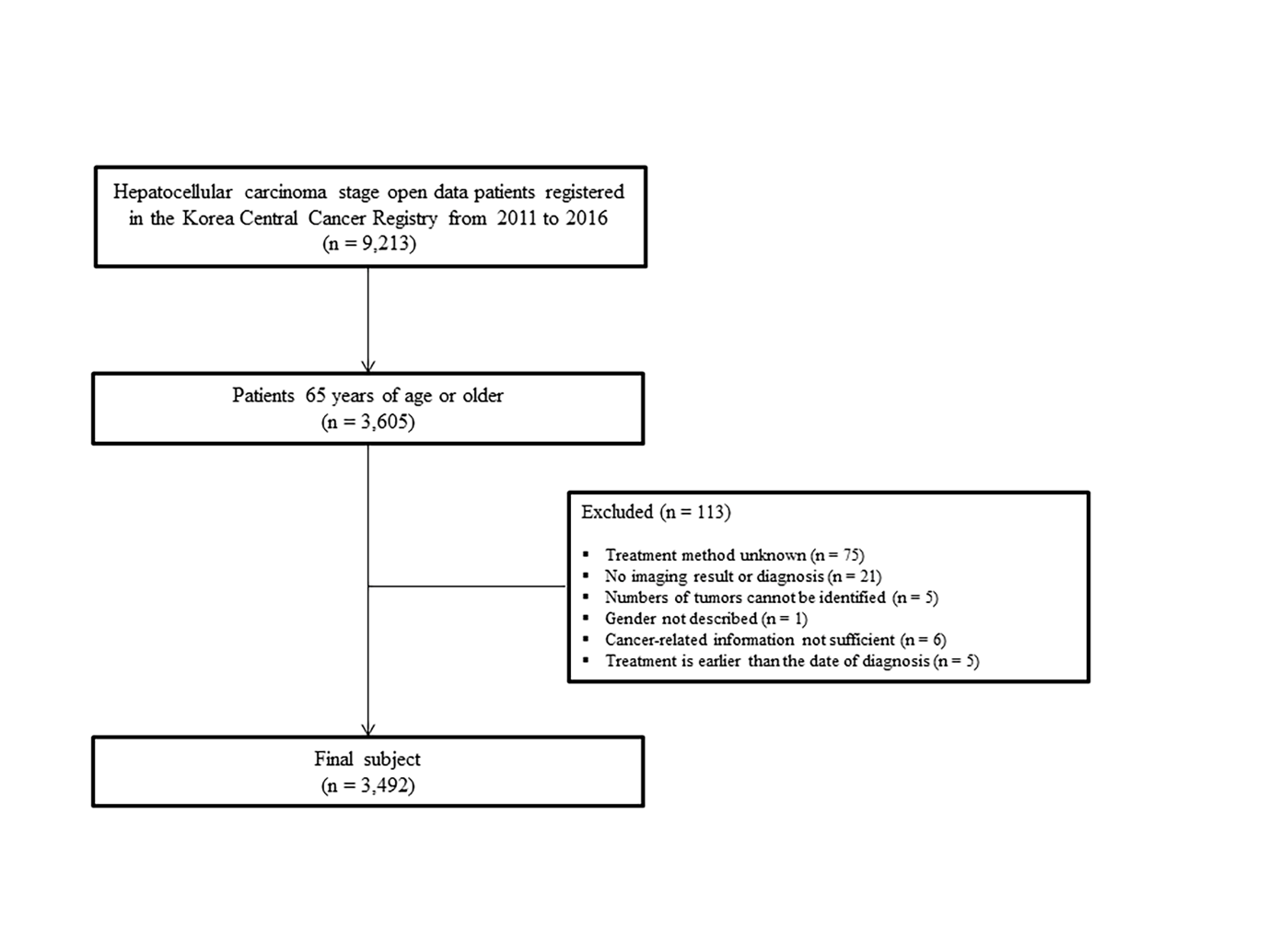


**Supplementary Fig 3.** **Flowchart displaying the selection process of patients**
