# Supplementary material for: A nationwide study on the current treatment status and natural prognosis of hepatocellular carcinoma in elderly
Source: Sci Rep. 2023 Sep 4;13:14584. doi: 10.1038/s41598-023-41771-5 (PMC10477316; doi:10.1038/s41598-023-41771-5)
Supplement: Supplementary file 2 — Supplementary Tables. [file 41598_2023_41771_MOESM2_ESM.docx]

**Supplementary Table 1. Survival table**

(A) according to BCLC stage

|  | **Total** | **Death** | | **median survival time(year)** | **1-year** | | **3-year** | | **5-year** | |
| --- | --- | --- | --- | --- | --- | --- | --- | --- | --- | --- |
|  |  | **n** | **(%)** |  | **Survival rate** | **SE** | **Survival rate** | **SE** | **Survival rate** | **SE** |
| Stage 0 | 211 | 117 | (55.45) | 7.255 | 0.962 | 0.013 | 0.782 | 0.028 | 0.654 | 0.033 |
| Stage A | 740 | 388 | (52.43) | 5.915 | 0.926 | 0.010 | 0.732 | 0.016 | 0.573 | 0.019 |
| Stage B | 641 | 502 | (78.32) | 2.082 | 0.688 | 0.018 | 0.413 | 0.020 | 0.282 | 0.018 |
| Stage C | 1154 | 1,005 | (87.09) | 0.915 | 0.470 | 0.015 | 0.259 | 0.013 | 0.169 | 0.011 |
| Stage D | 219 | 211 | (96.35) | 0.249 | 0.178 | 0.026 | 0.078 | 0.018 | 0.041 | 0.013 |

(B) according to modified UICC stage

|  | **Total** | **Death** | | **median survival time(year)** | **1-year** | | **3-year** | | **5-year** | |
| --- | --- | --- | --- | --- | --- | --- | --- | --- | --- | --- |
|  |  | **n** | **(%)** |  | **Survival rate** | **SE** | **Survival rate** | **SE** | **Survival rate** | **SE** |
| Stage I | 457 | 262 | (57.33) | 5.584 | 0.895 | 0.014 | 0.696 | 0.022 | 0.543 | 0.024 |
| Stage II | 1402 | 884 | (63.05) | 4.334 | 0.822 | 0.010 | 0.601 | 0.013 | 0.453 | 0.014 |
| Stage III | 982 | 826 | (84.11) | 1.584 | 0.618 | 0.016 | 0.336 | 0.015 | 0.221 | 0.013 |
| Stage IV-A | 337 | 325 | (96.44) | 0.416 | 0.276 | 0.024 | 0.086 | 0.015 | 0.040 | 0.011 |
| Stage IV-B | 309 | 303 | (98.06) | 0.249 | 0.149 | 0.020 | 0.052 | 0.013 | 0.023 | 0.008 |

**Supplementary Table 2. Treatment selection according to comorbidities**

|  |  | Supportive care | | Resection | | RFA | | TACE | | Chemotherapy | | Radiation | | Radioembolization | | Transplant | | Total | p |
| --- | --- | --- | --- | --- | --- | --- | --- | --- | --- | --- | --- | --- | --- | --- | --- | --- | --- | --- | --- |
|  |  | n | % | n | % | n | % | n | % | n | % | n | % | n | % | n | % |  |  |
|  |  | 868 |  | 563 |  | 387 |  | 1431 |  | 176 |  | 38 |  | 20 |  | 9 |  | 3492 |  |
| Diabetes | |  |  |  |  |  |  |  |  |  |  |  |  |  |  |  |  |  |  |
|  | No | 554 | 24.73 | 383 | 17.1 | 250 | 11.16 | 905 | 40.4 | 106 | 4.73 | 25 | 1.12 | 10 | 0.45 | 7 | 0.31 | 2240 | 0.3363 |
|  | Yes | 314 | 25.08 | 180 | 14.38 | 137 | 10.94 | 526 | 42.01 | 70 | 5.59 | 13 | 1.04 | 10 | 0.8 | 2 | 0.16 | 1252 |  |
| Hypertension | |  |  |  |  |  |  |  |  |  |  |  |  |  |  |  |  |  |  |
|  | No | 422 | 26.31 | 224 | 13.97 | 186 | 11.6 | 669 | 41.71 | 77 | 4.8 | 14 | 0.87 | 7 | 0.44 | 5 | 0.31 | 1604 | 0.0351 |
|  | Yes | 446 | 23.62 | 339 | 17.96 | 201 | 10.65 | 762 | 40.36 | 99 | 5.24 | 24 | 1.27 | 13 | 0.69 | 4 | 0.21 | 1888 |  |

**Supplementary Table 3. Survival table according to treatment modality**

|  | **Total** | **Death** | | **median survival time(year)** | **1-year** | | **3-year** | | **5-year** | |
| --- | --- | --- | --- | --- | --- | --- | --- | --- | --- | --- |
|  |  | **n** | **(%)** |  | **Survival rate** | **SE** | **Survival rate** | **SE** | **Survival rate** | **SE** |
| **All patients** |  |  |  |  |  |  |  |  |  |  |
| Supportive care | 868 | 825 | 95.05 | 0.416 | 0.274 | 0.015 | 0.126 | 0.011 | 0.065 | 0.008 |
| Surgical resection | 563 | 233 | 41.39 | 8.164 | 0.922 | 0.011 | 0.801 | 0.017 | 0.692 | 0.020 |
| RFA | 387 | 193 | 49.87 | 6.669 | 0.923 | 0.014 | 0.780 | 0.021 | 0.619 | 0.025 |
| TACE | 1431 | 1131 | 79.04 | 2.663 | 0.779 | 0.011 | 0.449 | 0.013 | 0.290 | 0.012 |
| Chemotherapy | 176 | 170 | 96.59 | 0.497 | 0.233 | 0.032 | 0.063 | 0.018 | 0.034 | 0.014 |
| Radiation therapy | 38 | 35 | 92.11 | 0.621 | 0.342 | 0.077 | 0.132 | 0.055 | 0.079 | 0.044 |
| Radioembolization | 20 | 14 | 70 | 2.918 | 0.900 | 0.067 | 0.450 | 0.111 | 0.300 | 0.103 |
| Liver transplant | 9 | 4 | 44.44 | . | 0.778 | 0.139 | 0.667 | 0.157 | 0.667 | 0.157 |
| **BCLC stage O or A** |  |  |  |  |  |  |  |  |  |  |
| Supportive care | 52 | 39 | 75 | 3.500 | 0.712 | 0.063 | 0.539 | 0.069 | 0.299 | 0.065 |
| Surgical resection | 242 | 77 | 31.82 | . | 0.967 | 0.012 | 0.888 | 0.020 | 0.796 | 0.026 |
| RFA | 243 | 111 | 45.68 | 7.504 | 0.959 | 0.013 | 0.823 | 0.025 | 0.682 | 0.030 |
| TACE | 404 | 271 | 67.08 | 4.671 | 0.928 | 0.013 | 0.641 | 0.024 | 0.462 | 0.025 |
| Chemotherapy | 4 | 2 | 50 | . | 1.000 | 0.000 | 0.500 | 0.250 | 0.500 | 0.250 |
| Radiation therapy | 4 | 3 | 75 | 3.500 | 0.750 | 0.217 | 0.500 | 0.250 | . | . |
| Radioembolization | 2 | 2 | 100 | 2.711 | 1.000 | 0.000 | 0.500 | 0.354 | 0.000 | . |
| **BCLC stage B** |  |  |  |  |  |  |  |  |  |  |
| Supportive care | 151 | 145 | 96.03 | 0.660 | 0.371 | 0.039 | 0.159 | 0.030 | 0.059 | 0.019 |
| Surgical resection | 131 | 61 | 46.56 | 7.669 | 0.878 | 0.029 | 0.733 | 0.039 | 0.621 | 0.043 |
| RFA | 6 | 5 | 83.33 | 3.834 | 0.833 | 0.152 | 0.667 | 0.193 | 0.333 | 0.193 |
| TACE | 315 | 262 | 83.17 | 2.334 | 0.771 | 0.024 | 0.419 | 0.028 | 0.254 | 0.025 |
| Chemotherapy | 18 | 17 | 94.44 | 0.706 | 0.333 | 0.111 | 0.056 | 0.054 | 0.056 | 0.054 |
| Radiation therapy | 5 | 5 | 100 | 0.753 | 0.400 | 0.219 | 0.000 | . | 0.000 | . |
| Radioembolization | 12 | 6 | 50 | . | 0.917 | 0.080 | 0.500 | 0.144 | 0.500 | 0.144 |
| Liver transplant | 3 | 1 | 33.33 | . | 1.000 | 0.000 | 0.667 | 0.272 | 0.667 | 0.272 |
| **BCLC stage C** |  |  |  |  |  |  |  |  |  |  |
| Supportive care | 388 | 375 | 96.65 | 0.332 | 0.196 | 0.020 | 0.083 | 0.014 | 0.040 | 0.010 |
| Surgical resection | 104 | 54 | 51.92 | 7.003 | 0.894 | 0.030 | 0.721 | 0.044 | 0.610 | 0.048 |
| RFA | 61 | 34 | 55.74 | 5.663 | 0.869 | 0.043 | 0.771 | 0.054 | 0.525 | 0.066 |
| TACE | 436 | 381 | 87.39 | 1.792 | 0.654 | 0.023 | 0.310 | 0.022 | 0.184 | 0.019 |
| Chemotherapy | 139 | 136 | 97.84 | 0.416 | 0.201 | 0.034 | 0.058 | 0.020 | 0.022 | 0.012 |
| Radiation therapy | 24 | 23 | 95.83 | 0.581 | 0.250 | 0.088 | 0.083 | 0.056 | 0.042 | 0.041 |
| Radioembolization | 2 | 2 | 100 | 0.918 | 0.500 | 0.354 | 0.000 | . | 0.000 | . |

**Supplementary Table 4. Factors affecting overall mortality in elderly patients with hepatocellular carcinoma (≥ 75 years)**

| **Variables** | **Univariable** | | **Multivariable** | |
| --- | --- | --- | --- | --- |
|  | HR (95% CI) | *P* | HR (95% CI) | *P* |
| **Treatment option (first-line)** |  |  |  |  |
| Supportive care | 1 (ref) |  | 1 (ref) |  |
| Surgical resection | 0.147 (0.112-0.194) | <0.001 | 0.259 (0.193-0.349) | <0.001 |
| RFA | 0.166 (0.128-0.215) | <0.001 | 0.298 (0.233-0.399) | <0.001 |
| TACE | 0.340 (0.297-0.389) | <0.001 | 0.482 (0.411-0.566) | <0.001 |
| Systemic chemotherapy | 1.233 (0.964-1.578) | 0.0955 | 1.017 (0.772-1.340) | 0.9045 |
| Radiation therapy | 1.037 (0.629-1.707) | 0.8878 | 1.037 (0.607-1.771) | 0.8944 |
| Radioembolization | 0.263 (0.131-0.530) | 0.0002 | 0.392 (0.173-0.888) | 0.0247 |
| **Male sex (vs. female)** | 0.968 (0.855-1.095) | 0.604 | 0.981 (0.852-1.129) | 0.7903 |
| **High BMI (≥ 25 kg/m^2^)** | 0.776 (0.673-0.896) | 0.0005 | 0.754 (0.649-0.877) | 0.0002 |
| **Diabetes** | 1.133 (1.005-1.277) | 0.0415 | 1.170 (1.025-1.336) | 0.0200 |
| **Hypertension** | 0.944 (0.868-1.062) | 0.3363 | 1.009 (0.883-1.153) | 0.8955 |
| **ECOG performance status** |  |  |  |  |
| Status 0 | 1 (ref) |  | 1 (ref) |  |
| Status 1 | 1.599 (1.336-1.913) | <0.0001 | 1.124 (0.929-1.361) | 0.2290 |
| Status 2 | 2.186 (1.728-2.764) | <0.0001 | 1.219 (0.948-1.567) | 0.1222 |
| Status 3 | 3.142 (2.275-4.341) | <0.0001 | 1.102 (0.767-1.584) | 0.5996 |
| Status 4 | 8.597 (5.483-13.424) | <0.0001 | 3.544 (2.191-5.732) | <0.0001 |
| **HBsAg-positive** | 0.925 (0.796-1.076) | 0.3149 | 0.967 (0.812-1.152) | 0.7066 |
| **Anti-HCV positive** | 1.003 (0.891-1.152) | 0.9655 | 1.034 (0.881-1.214) | 0.6802 |
| **Ascites** |  |  |  |  |
| None | 1 (ref) |  |  |  |
| Mild | 2.142 (1.835-2.495) | <0.0001 |  |  |
| Moderate to severe | 3.515 (2.914-4.239) | <0.0001 |  |  |
| **Child-pugh class** |  |  |  |  |
| Class A | 1 (ref) |  | 1 (ref) |  |
| Class B | 2.479 (2.169-2.833) | <0.0001 | 1.617 (1.379-1.898) | <0.0001 |
| Class C | 6.092 (4.629-8.018) | <0.0001 | 3.200 (2.323-4.408) | <0.0001 |
| **MELD score** |  |  |  |  |
| < 10 | 1 (ref) |  | 1 (ref) |  |
| ≥ 10 | 1.920 (1.699-2.171) | <0.0001 | 1.189 (1.031-1.372) | 0.0174 |
| **Number of tumors** |  |  |  |  |
| 1 | 1 (ref) |  | 1 (ref) |  |
| More than 2 | 1.748 (1.551-1.969) | <0.0001 | 1.261 (1.033-1.538) | 0.0224 |
| **Size of tumors** |  |  |  |  |
| < 3cm | 1 (ref) |  | 1 (ref) |  |
| ≥ 3cm | 1.886 (1.647-2.161) | <0.0001 | 1.438 (1.204-1.716) | <0.0001 |
| **Portal vein invasion** | 2.674 (2.334-3.064) | <0.0001 | 1.445 (1.155-1.807) | 0.0013 |
| **Modified UICC stage** |  |  |  |  |
| Stage I | 1 (ref) |  | 1 (ref) |  |
| Stage II | 1.171 (0.935-1.466) | 0.1691 | 0.789 (0.606-1.029) | 0.0799 |
| Stage III | 2.135 (1.699-2.681) | <0.0001 | 0.907 (0.643-1.280) | 0.5789 |
| Stage IV-A | 4.415 (3.371-5.782) | <0.0001 | 1.221 (0.765-1.950) | 0.4029 |
| Stage IV-B | 6.396 (4.900-8.347) | <0.0001 | 1.597 (1.084-2.354) | 0.0179 |
| **BCLC stage** |  |  |  |  |
| Stage 0 | 1 (ref) |  | 1 (ref) |  |
| Stage A | 0.870 (0.606-1.249) | 0.4514 |  |  |
| Stage B | 1.811 (1.278-2.566) | 0.0008 |  |  |
| Stage C | 2.738 (1.953-3.839) | <0.0001 |  |  |
| Stage D | 6.074 (4.159-8.871) | <0.0001 |  |  |
| **Serum albumin** |  |  |  |  |
| < 4 g/dL | 1 (ref) |  | 1 (ref) |  |
| ≥ 4 g/dL | 0.461 (1.501-1.903) | <0.0001 |  |  |
| **Total bilirubin** |  |  |  |  |
| < 1 mg/dL | 1 (ref) |  | 1 (ref) |  |
| ≥ 1 mg/dL | 1.690 (1.501-1.903) | <0.0001 |  |  |
| **Platelet (x 10^3^/ uL)** |  |  |  |  |
| ≥ 150 | 1 (ref) |  | 1 (ref) |  |
| < 150 | 1.110 (0.986-1.249) | 0.0846 | 1.037 (0.901-1.194) | 0.6091 |

* HR, hazard ratio; CI, confidence interval; RFA, radiofrequency ablation; TACE, trasarterial chemoembolization; BMI, body mass index; ECOG, Eastern Cooperative Oncology Group; HBsAg, hepatitis B virus surface antigen; HCV, hepatitis C virus; MELD, Model For End-Stage Liver Disease; UICC, The Union for International Cancer Control; BCLC, Barcelona Clinic Liver Cancer

**Supplementary Table 5. Survival table of untreated elderly hepatocellular carcinoma**

|  |  | **Total** | **Death** | | **median survival time(year)** | **1-year** | | **3-year** | | **5-year** | |
| --- | --- | --- | --- | --- | --- | --- | --- | --- | --- | --- | --- |
|  |  |  | **n** | **(%)** |  | **Survival rate** | **SE** | **Survival rate** | **SE** | **Survival rate** | **SE** |
| All patients |  | 868 | 825 | 95.05 | 0.416 | 0.274 | 0.015 | 0.126 | 0.011 | 0.065 | 0.008 |
| ≥ 65 years | Stage 0 | 12 | 8 | 66.67 | 3.792 | 0.917 | 0.080 | 0.833 | 0.108 | 0.417 | 0.142 |
|  | Stage A | 40 | 31 | 77.5 | 2.334 | 0.650 | 0.075 | 0.450 | 0.079 | 0.259 | 0.072 |
|  | Stage B | 151 | 145 | 96.03 | 0.660 | 0.371 | 0.039 | 0.159 | 0.030 | 0.059 | 0.019 |
|  | Stage C | 388 | 375 | 96.65 | 0.332 | 0.196 | 0.020 | 0.083 | 0.014 | 0.040 | 0.010 |
|  | Stage D | 153 | 150 | 98.04 | 0.241 | 0.065 | 0.020 | 0.026 | 0.013 | 0.020 | 0.011 |
| ≥ 75 years | Stage 0 | 5 | 4 | 80 | 3.666 | 1.000 | 0.000 | 0.800 | 0.179 | 0.200 | 0.179 |
|  | Stage A | 19 | 16 | 84.21 | 0.830 | 0.474 | 0.115 | 0.368 | 0.111 | 0.190 | 0.094 |
|  | Stage B | 99 | 96 | 96.97 | 0.501 | 0.333 | 0.047 | 0.121 | 0.033 | 0.027 | 0.017 |
|  | Stage C | 201 | 194 | 96.52 | 0.249 | 0.214 | 0.029 | 0.080 | 0.019 | 0.045 | 0.015 |
|  | Stage D | 84 | 83 | 98.81 | 0.167 | 0.083 | 0.030 | 0.024 | 0.017 | . | . |
